# Inhibition of PCSK9 enhances the antitumor effect of PD-1 inhibitor in colorectal cancer by promoting the infiltration of CD8^+^ T cells and the exclusion of Treg cells

**DOI:** 10.3389/fimmu.2022.947756

**Published:** 2022-08-08

**Authors:** Rui Wang, Hongchuan Liu, Peng He, Duopeng An, Xiaohan Guo, Xuyao Zhang, Meiqing Feng

**Affiliations:** Department of Biological Medicines & Shanghai Engineering Research Center of Immunotherapeutics, Fudan University School of Pharmacy, Shanghai, China

**Keywords:** PD-1, CD8^+^ T cells, regulatory T cells, tumor microenvironment, PCSK9

## Abstract

Immunotherapy especially immune checkpoint inhibitors (ICIs) has brought favorable clinical results for numerous cancer patients. However, the efficacy of ICIs in colorectal cancer (CRC) is still unsatisfactory due to the poor median progression-free survival and overall survival. Here, based on the CRC models, we tried to elucidate novel relapse mechanisms during anti-PD-1 therapy. We found that PD-1 blockade elicited a mild antitumor effect in these tumor models with both increased CD8^+^ T cells and Treg cells. Gene mapping analysis indicated that proprotein convertase subtilisin/kexin type 9 (PCSK9), low-density lipoprotein receptor, transforming growth factor-β (TGF-β), and CD36 were unexpectedly upregulated during PD-1 blockade. To investigate the critical role of these proteins especially PCSK9 in tumor growth, anti-PCSK9 antibody in combination with anti-PD-1 antibody was employed to block PCSK9 and PD-1 simultaneously in CRC. Data showed that neutralizing PCSK9 during anti-PD-1 therapy elicited a synergetic antitumor effect with increased CD8^+^ T-cell infiltration and inflammatory cytokine releases. Moreover, the proportion of Treg cells was significantly reduced by co-inhibiting PCSK9 and PD-1. Overall, inhibiting PCSK9 can further enhance the antitumor effect of anti-PD-1 therapy in CRC, indicating that targeting PCSK9 could be a promising approach to potentiate ICI efficacy.

## Introduction

Colorectal cancer (CRC) is the second leading cause of cancer-related death with an incidence of 10.2% and a mortality of 9.2% (1, 2). As a promising treatment to modulate the host’s immune system, immunotherapy such as immune checkpoint inhibitors (ICIs) shows durable antitumor effects and revolutionizes the management of various cancers including melanoma and non-small cell lung cancer (NSCLC) (3, 4). However, the low response rate and emerging resistance mechanism still pose limitations to the application of ICIs in CRC (5). Therefore, studies dedicated to overcoming CRC resistance to ICIs are in urgent need.

Studies have uncovered that tumor microenvironment (TME) consisting of various components plays a critical role during antitumor immunity induced by ICIs. Infiltration of immune cells and the interaction between immune cells and tumor cells posed a significant impact on the outcomes of ICI therapy (6). Furthermore, the nutrient-deficient and hypoxic microenvironment also force immune cells to undergo metabolic transformation to immune-tolerant phenotypes. Metabolic regulation of glucose, lactate, and especially lipid can refuel immune cells to favor antitumor immunity in TME (7, 8). Glycolysis induced by autophagy was indispensable for oncogenic transformation (9). Our previous research also demonstrated that inhibiting autophagy could enhance phagocytosis and cytotoxicity of macrophages and further potentiate the antitumor effect of ICIs (10, 11). Cholesterol accumulation in TME facilitates the polarization and activity of tumor-associated macrophages (TAMs) and further impairs cytokine release of CD8^+^ T cells (12, 13). These studies indicate that targeting metabolism in TME could be a potential modality to potentiate the efficacy of ICIs.

Recently, proprotein convertase subtilisin/kexin type 9 (PCSK9), a lipid metabolism-related protein, has been reported to be critical for tumorigenesis and progression (14). PCSK9 is an indispensable element in regulating lipid metabolism by inducing the degradation of low-density lipoprotein receptor (LDLR) in lysosome. Inhibitors of PCSK9 have been approved for the treatment of atherosclerotic cardiovascular diseases associated with hypercholesterolemia (15, 16). More importantly, PCSK9 has been proven to disrupt the recycling of MHC I to the cell surface by promoting MHC I degradation. Inhibiting PCSK9 by small molecular compounds or monoclonal antibodies increases the expression of MHC I on the tumor cell surface, promoting intratumoral infiltration of cytotoxic lymphocytes (17, 18). These data reveal that PCSK9 may be a crucial regulator for cancer immunotherapy. However, the effects of PCSK9 in CRC under anti-PD-1 therapy are still unclear. Hence, in this study, two syngeneic CRC models were constructed to elucidate the crucial role and mechanism of PCSK9 during anti-PD-1 therapy.

## Results

### PD-1 blockade presented a mild antitumor effect with increased CD8^+^ T cells and Treg cells

**Figure 1:**
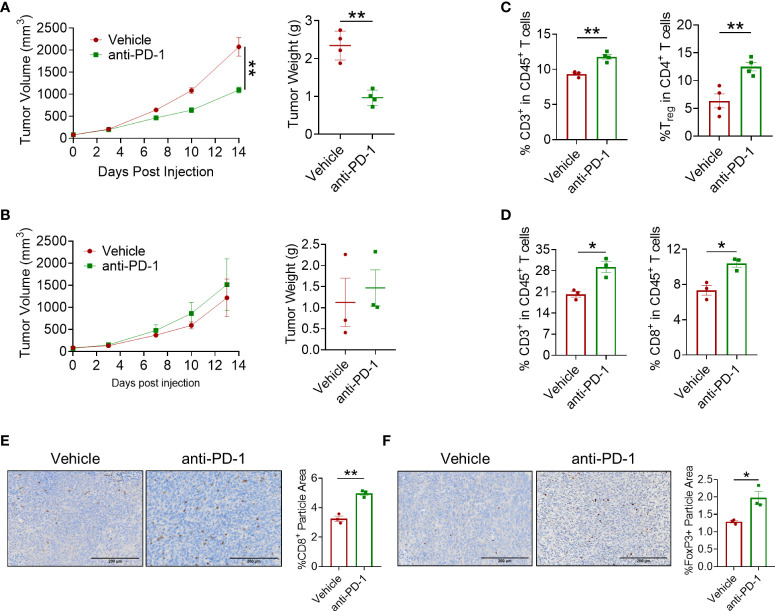
*In vivo* antitumor effect of anti-PD-1 antibody. **(A)** Tumor volume and tumor weight in the MC38 tumor model. Anti-PD-1 mAb administration was 5 mg/kg, *n* = 4 mice/group. **(B)** Tumor volume and tumor weight of CT26 tumor model. Anti-PD-1 mAb administration was 5 mg/kg, *n* = 3 mice/group. **(C, D)** Flow cytometry analysis of tumor-infiltrating T cells for mice treated with PBS or anti-PD-1 antibody in MC38 tumor model **(C)** and in CT26 tumor model **(D)**. **(E, F)** IHC staining of CD8a and Foxp3 in CT26 tumors. "*" means p-value < 0.05 and "**" means p-value < 0.01.

To examine the effect of PD-1 blockade in CRC, MC38 and CT26 tumor models were well-constructed and anti-PD-1 antibody was administered at a dose of 5 mg/kg twice a week. In the MC38 tumor model, results showed that PD-1 blockade exerted a mild antitumor effect as the tumor growth was delayed 14 days after the first administration (**Figure 1A**). However, no antitumor effect of the anti-PD-1 antibody was observed in the CT26 tumor model (**Figure 1B**). In view of the mild efficacy of anti-PD-1 therapy in CRC, we detected the infiltration of CD8^+^ T cells in the tumors. Flow cytometry analysis showed that CD45^+^CD3^+^ T lymphocytes and CD8^+^ T cells were significantly increased in both CRC models (**Figures 1C, D**). However, regulatory T (Treg) cells were also elevated by PD-1 blockade in these models (**Figure 1C**). Furthermore, IHC staining confirmed that PD-1 blockade increased both CD8^+^ T lymphocytes and Foxp3^+^ cells in the tumors (**Figures 1E, F**). These results indicated that anti-PD-1 antibody elicited a mild antitumor effect in CRC with increased infiltration of Tregs and CD8^+^ T cells.

### PD-1 blockade enhanced PCSK9 expression

**Figure 2:**
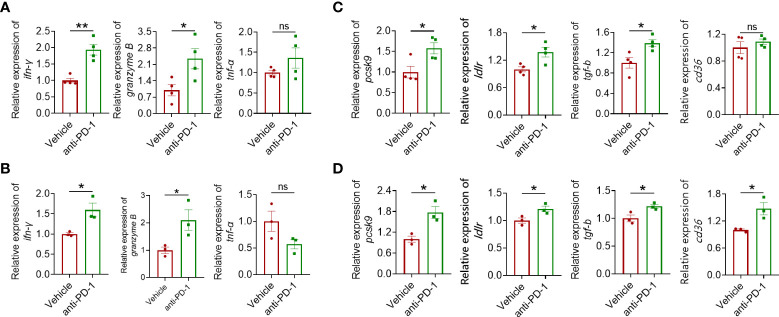
Quantitative real-time PCR (qRT-PCR) analysis of IFN-γ, granzyme B, and TNF-α gene expression in MC38 tumors **(A)** and CT26 tumors **(B)**. RT-qPCR analysis of PCSK9, LDLR, TGF-β, and CD36 gene expression in tumor of the MC38 tumor model **(C)** and the CT26 tumor model **(D)**. "*" means p-value < 0.05 and "**" means p-value < 0.01 while "ns" means not statistically significant.

To further explore the underlying mechanism leading to the mild antitumor effect of anti-PD-1 ICI in CRC, several landmark cytokines of immune cell cytotoxicity including IFN-γ, granzyme B, and TNF-α were evaluated. As shown in **Figure 2**, IFN-γ and granzyme B in CRC models were upregulated after blocking PD-1 while the level of TNF-α was barely affected (**Figures 2A, B**). Except for these inflammation-related cytokines, we found that anti-PD-1 therapy also engaged in the regulation of lipid metabolism-related proteins including PCSK9 and LDLR (**Figures 2C, D**). LDLR is a pivotal receptor in cholesterol regulation, which is targeted by PCSK9. When binding to LDLR, PCSK9 can promote its degradation in lysosome (19). Interestingly, the mRNA level of CD36 was upregulated in CT26 tumors but not in MC38 tumors. In summary, PD-1 blockade showed a significant influence on the gene expression of lipid metabolism-related proteins including PCSK9 in colorectal tumors.

### Co-targeting PD-1 and PCSK9 elicited an enhanced antitumor effect

**Figure 3:**
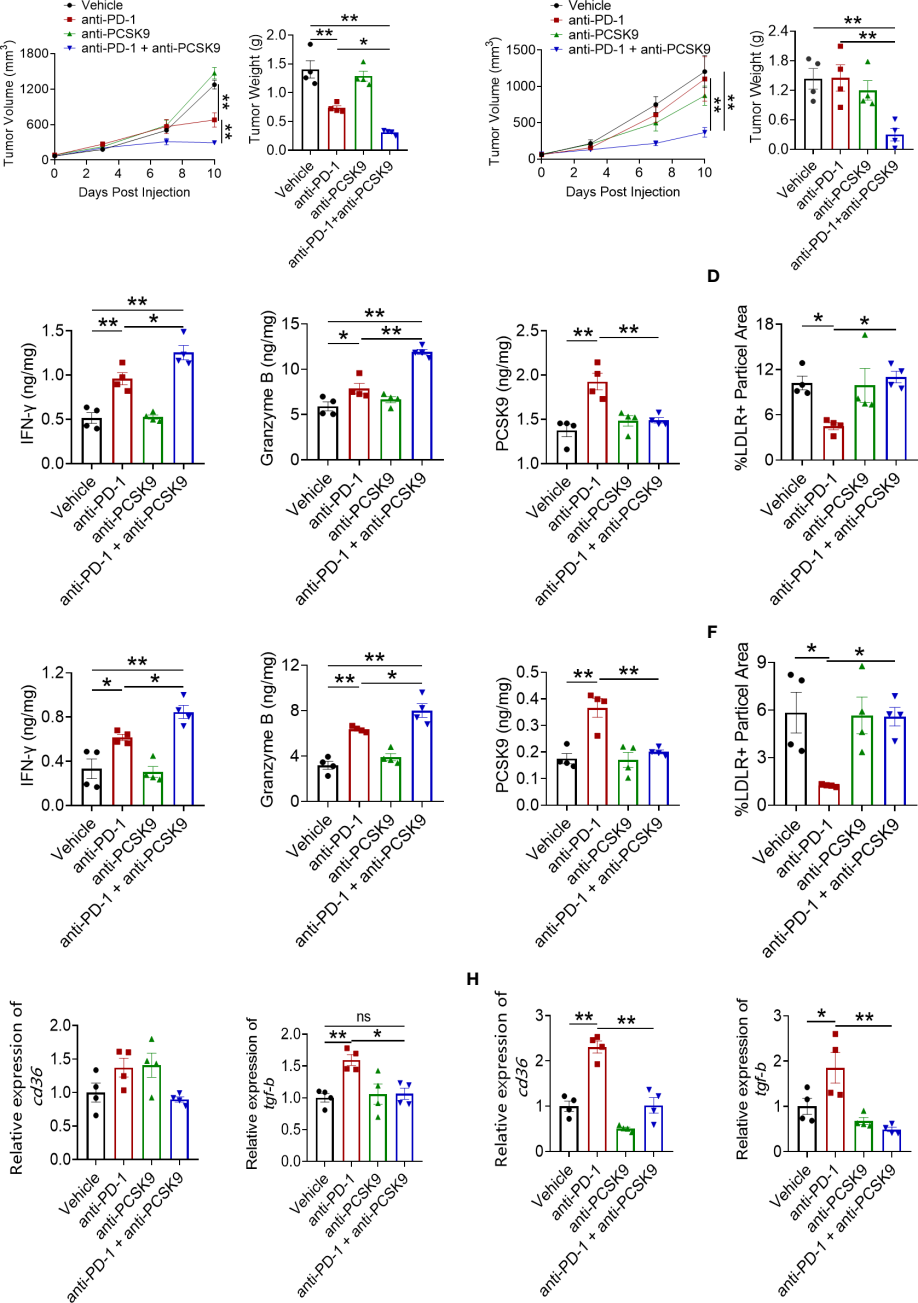
Tumor volume and tumor weight in MC38 tumor model **(A)** and CT26 tumor model **(B)** treated with anti-PD-1 or anti-PCSK9 antibody. Analysis of IFN-γ, granzyme B and PCSK9 protein level in tumor of MC38 tumor model **(C)** and CT26 tumor model **(E)** by ELISA. Quantitative analysis of LDLR expression on cell membrane in MC38 tumors **(D)** and CT26 tumors **(F)** by immunofluorescence. Quantitative RT-PCR analysis of CD36 and TGF-β gene expression in MC38 tumors **(G)** and in CT26 tumors **(H)**. "*" means p-value < 0.05 and "**" means p-value < 0.01 while "ns" means not statistically significant.

Considering the enhanced PCSK9 expression during anti-PD-1 therapy, anti-PCSK9 antibody was employed to detect the critical role of PCSK9 in CRC. **Figure 3A** shows that anti-PCSK9 antibody further potentiated the antitumor effect of PD-1 inhibitor in the MC38 tumor model. In the CT26 tumor model, PD-1 inhibitor in combination with anti-PCSK9 antibody elicited synergetic antitumor effects while PD-1 blockade or anti-PCSK9 alone displayed indiscernible effects on the tumor growth (**Figure 3B**). PD-1 ICI in combination with anti-PCSK9 antibody showed enhanced antitumor effects in CRC models (**Figures 3A, B**), but a similar effect was not observed in a breast cancer model (data not shown). On D10 after administration, tumors were excised to detect the level of IFN-γ, TNF-α, and granzyme B. In MC38 tumors, anti-PD-1 antibody and anti-PCSK9 antibody co-treatment led to significant increases in granzyme B and IFN-γ (**Figure 3C**, **Supplementary Figure 1A**). Then, we analyzed the level of PCSK9, LDLR, TGF-β, and CD36. Compared to PD-1 inhibitor alone, anti-PCSK9 antibody diminished the increased expression of PCSK9, LDLR, TGF-β, and CD36 (**Figures 3C–H**, **Supplementary Figures 1B–E**). These data indicated that targeting PD-1 and PCSK9 elicited a synergetic antitumor effect in CRC.

### Anti-PCSK9 promoted CD8^+^ T-cell infiltration induced by PD-1 inhibitor

**Figure 4:**
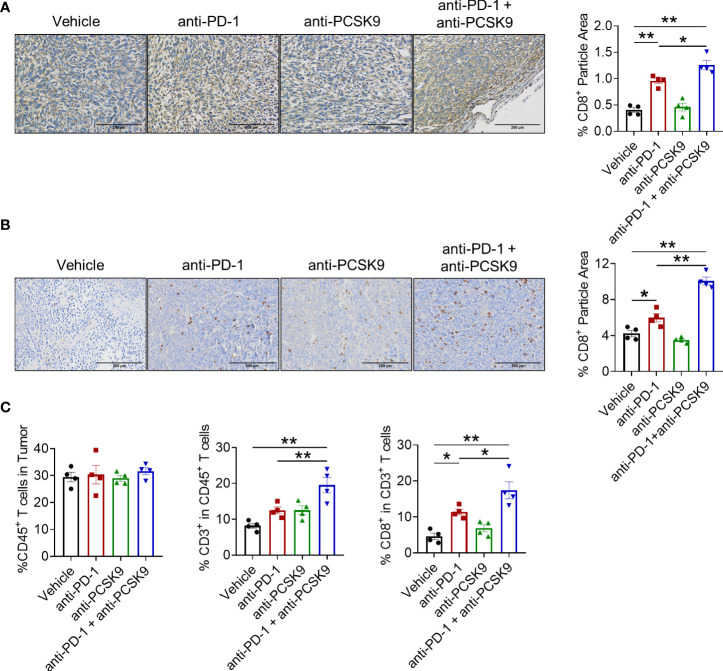
**(A)** IHC staining of CD8a in tumor of the MC38 tumor model treated with anti-PD-1 or anti-PCSK9 antibody and quantitative analysis of positive particles and **(B)** for the CT26 tumor model. **(C)** Flow cytometry analysis of CD45^+^, CD3^+^, and CD8^+^ T-cell infiltration in MC38 tumors. "*" means p-value < 0.05 and "**" means p-value < 0.01.

To explore the synergetic antitumor effect of anti-PD-1 and anti-PCSK9 antibodies in CRC, tumor-infiltrating CD8^+^ T cells were detected. IHC staining showed that PD-1 inhibition led to the increased tumor-infiltrating CD8^+^ T cells in MC38 and CT26 tumor models. Despite no significant elevation of CD8^+^ T cells induced by PCSK9 blockade alone, PD-1 inhibitor combined with anti-PCSK9 antibody indeed potentiated the infiltration of CD8^+^ T cells (**Figures 4A, B**). In addition, flow cytometry analysis showed that the proportion of tumor-infiltrating CD8^+^ T cells in the combination therapy was obviously higher than either monotherapy group (**Figure 4C**). These data demonstrated that targeting PCSK9 potentiated the antitumor effect of PD-1 blockade *via* promoting the infiltration of cytotoxic CD8^+^ T cells.

### PCSK9 blockade eliminated the increased Treg cells induced by PD-1 inhibitor

**Figure 5:**
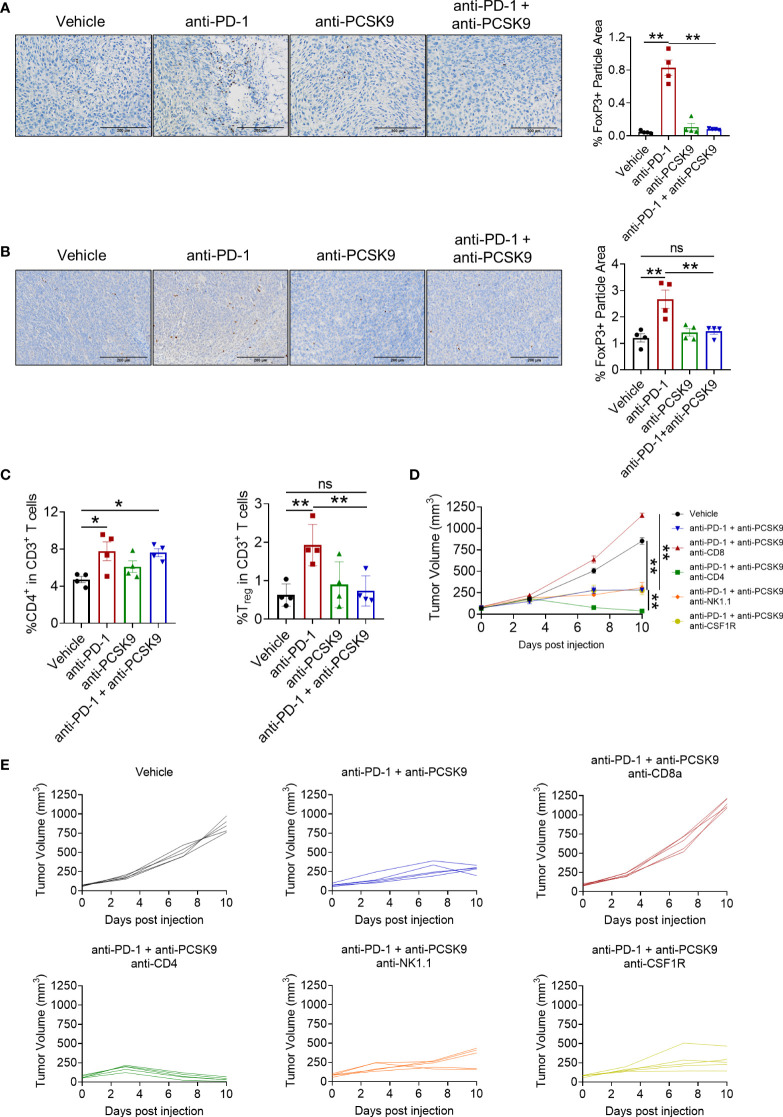
**(A)** IHC staining of Foxp3 in MC38 tumors treated with anti-PD-1 or anti-PCSK9 antibody and quantitative analysis of positive particles and **(B)** for CT26 tumors. **(C)** Flow cytometry analysis of CD4^+^ and Foxp3^+^ T-cell infiltration in MC38 tumors. **(D, E)** Tumor volume in the mice under the treatment of anti-PD-1 and anti-PCSK9 antibody with/without anti-CD8, anti-CD4, anti-NK1.1, and anti-CSF1R antibody. "*" means p-value < 0.05 and "**" means p-value < 0.01 while "ns" means not statistically significant.

Treg cell is a typical suppressive immune cell, promoting tumor cells to escape from immune surveillance. We next detected whether anti-PCSK9 antibody affected PD-1 blockade-induced Treg cells *via* IHC analysis. Compared with PD-1 blockade, anti-PCSK9 antibody alone has no obvious effect on Treg cells, while anti-PCSK9 antibody combined with anti-PD-1 antibody eliminated the increased Treg cells (**Figures 5A, B**). Furthermore, flow cytometry further confirmed that the PD-1 inhibitor-increased percentage of Treg cell proportion was decreased by anti-PCSK9 antibody (**Figure 5C**). Except for CD8^+^ T cells and Treg cells, we did not observe significant influence on innate immune cells including macrophages, natural killer cells, and dendritic cells by simultaneous inhibition of PD-1 and PCSK9 (**Supplementary Figures 2A, B**).

Finally, to verify which lymphocyte subpopulation contributed to the synergistic antitumor effect of targeting PD-1 and PCSK9 in CRC, anti-CD4, anti-CD8, anti-NK1.1, and anti-CSF1R antibody were applied to deplete the corresponding subset of immune cells, respectively. These antibodies have been proved to block the cells with relevant marker in mouse spleen (**Supplementary Figure 2C**). As shown in **Figures 5D, E**, CD8^+^ T depletion totally eliminated the antitumor effect of targeting PD-1 and PCSK9. Depletion of NK cells or macrophages barely affected the tumor burden. Importantly, CD4^+^ T cell-depleting antibody presented an unexpected enhancement on the antitumor effect of anti-PD-1 and anti-PCSK9 cotreatment. Overall, our results indicated that PCSK9 blockade enhanced the antitumor effect of PD-1 inhibitor through eliminating the increased Treg cells.

## Discussion

Since the discovery of ICIs, the therapeutic paradigm of cancer has been changed remarkably. However, the antitumor efficacy of ICIs in solid tumor is still unsatisfied although it has achieved tumor remission in some patients. Compared with melanoma or NSCLC, the objective response rate of ICI therapy in CRC patients is much lower (20). Currently, because of the extensive application of ICIs in clinic, many strategies attempt to overcome resistance to ICIs. The combination of different ICIs or ICIs with proinflammatory cytokines has been proposed to further enhance antitumor immune response. Simultaneous administration of immune-enhancing agents can indeed improve antitumor immunity but is accompanied by a higher risk of immune-related adverse effects (21). Triple combination therapy, such as anti-PD-L1 antibody, poly-(ADP-ribose) polymerase inhibitor, and MEK inhibitor, was also applied to overcome resistance to anti-PD-L1 therapies in KRAS mutant cancer (22). Agonists targeting STING in combination with CTLA-4 or PD-1 inhibitor also exerted refreshing efficacy in the CRC model with complete tumor regression and long-lasting immune memory (23). In this study, we investigated PCSK9 during anti-PD-1 therapy in CRC models, and PD-1 inhibitor and anti-PCSK9 antibody were administered to confirm the synergetic antitumor effect of targeting PD-1 and PCSK9 in CRC.

In syngeneic CRC models, PD-1 inhibitor only has a limited antitumor effect with the infiltration of CD8^+^ T cells and Tregs. As a type of immunosuppressive T cell, Treg is indispensable in maintaining normal tissue homeostasis by restraining excessive immune responses. However, the immunosuppressive effect of Tregs also facilitates tumor cells to avoid immune surveillance (24). Studies have indicated that Tregs performed immunosuppressive function through multifaceted ways including repressing the production of CD8^+^ T cell-derived IFN-γ and converting ATP to adenosine (25, 26). In our research, we found that PCSK9 blockade could eliminate increased tumor-infiltrating Tregs induced by PD-1 inhibitor. As a result, the antitumor effect of PD-1 blockade was significantly potentiated after Treg exclusion.

TGF-β signaling potentiates the immunosuppressive activity of Tregs, leading to a poor outcome of PD-L1 blockade, and TGF-β neutralization could help overcome the resistance to ICIs (27–29). A previous study uncovered that PCSK9 deficiency could reduce SMAD2 phosphorylation and further promote TGFβR1 degradation in lysosome (30), indicating the internal relation between PCSK9 and TGF-β in TME. Consistent with these concepts, we observed the upregulation of TGF-β expression during anti-PD-1 therapy, which could be diminished by the administration of anti-PCSK9 antibody. Furthermore, studies indicated that expression of CD36, a type of scavenger receptor, was elevated on tumor-infiltrating Tregs and CD8^+^ T cells (31). In Tregs, CD36 could facilitate lipid uptake and stimulate mitochondrial fitness to maintain cell survival and proliferation, while CD36 enhanced oxidized low-density lipoprotein uptake and induced an unfavorable metabolic reprogramming in CD8^+^ T cells (32, 33). In this work, we confirmed that PD-1 blockade induced CD36 expression in CRC tumors and PSCK9 deficiency could eliminate the increased CD36.

In summary, the efficacy of PD-1 inhibitor was related to the expression level of PCSK9 in CRC. PCSK9-neutralizing antibody could enhance the antitumor effect of PD-1 inhibitor with increased CD8^+^ T-cell infiltration and Treg exclusion. Moreover, inhibiting PCSK9 could regulate lipid metabolism in CRC tumors *via* the downregulated expression of LDLR and CD36 to remodel TME to pro-inflammatory circumstance. Overall, our research proposed a novel idea to overcome ICI resistance in CRC by simultaneous inhibition of PD-1 and PCSK9.

## Materials and methods

### CRC cells and tumor models

Murine CRC cell lines MC38 and CT26 were cultured as described in previous articles (34, 35). Mice were provided by Shanghai Slack Laboratory Animal Co., Ltd. (Shanghai, China) and the animal experiments were approved by the Animal Ethical Committee of School of Pharmacy Fudan University. CRC tumor models were also well established according to the methods in the articles (34, 35). In brief, mice were randomly divided into the indicated groups. The formula for tumor volume was ½ × length × width^2^. Anti-PD-1 antibody monotherapy for MC38 and CT26 CRC models was injected (i.p.) twice a week at a dose of 5 mg/kg. Anti-PCSK9 antibody was injected (i.p.) twice a week at a dose of 10 mg/kg. Anti-CD8a, anti-CD4, antiNK1.1, and anti-CSF1R antibody was injected (i.p.) 1 day before antibody treatment at doses of 200 μg, 200 μg, 400 μg, and 300 μg per mouse, respectively.

### IHC staining

Tumor was fixed in formalin and then embedded with paraffin for section preparation. After the sections were dewaxed and hydrated, tissue antigen was repaired with citrate buffer. Endogenous peroxidase was deactivated using H_2_O_2_ and then blocked with BSA. The sections were incubated with primary antibody and HRP-labeled secondary antibodies, respectively. Then, these sections were counterstained with hematoxylin. Images were obtained by a microscope for analysis. The following were the antibodies used: rabbit anti-mouse CD8a antibody (Servicebio, GB11068), rabbit anti-mouse Foxp3 antibody (Servicebio, GB112325), rabbit anti-mouse CSF1R antibody (Servicebio, GB11581), rabbit anti-mouse NK1.1 antibody (abcam, ab289542), rabbit anti-mouse CD11b antibody (Servicebio, GB11581), and rabbit anti-mouse CD4 antibody (Servicebio, GB13064-2). Proportions of the positive area were counted by ImageJ software.

### IF staining

All operations were performed referring to a previous article (36). Materials used were as follows: DAPI (Servicebio, G1012) and rabbit anti-mouse LDLR antibody (Servicebio, GB11369). Proportions of the positive area were counted by ImageJ software.

### Flow cytometry analysis

Tumors were obtained to prepare single-cell suspension and then incubated with red blood cells lysis buffer. Cell density was adjusted to 1×10^6^ per milliliter. Cells were incubated with anti-CD16/32 antibody to block Fc receptors. After incubating with antibody targeted cell surface antigen including anti-45, anti-CD3, anti-CD8, anti-CD4, and anti-CD25 antibodies, cells were fixed with 1× Foxp3 Fix/Perm buffer and next incubated with 1× Foxp3 Perm buffer for the detection of intracellular antigen Foxp^3^. Finally, cells were incubated with anti-Foxp3 antibody in the dark at room temperature and analyzed with CytoFlex S (Beckman). The antibodies used were as follows: anti-CD45 antibody (MultiSciences, Violetflour 450, cat.70-AM04512-100; BioLegend, APC/Cyanine7, cat.103116), anti-CD3ϵ antibody (BioLegend, PE/Cyanine7, cat.100320; BioLegend, PerCP/Cyanine5.5, cat.100328), anti-CD4 antibody (BioLegend, APC, cat.100412), anti-CD8a antibody (BioLegend, PerCP, cat.100732; BioLegend, FITC, cat.100706), and anti-Foxp3 antibody (BioLegend, PE, cat.320008).

### ELISA

All the operations were carried out according to the manufacturer’ s instructions. ELISA kits used are as follows: IFN-γ ELISA kit (MultiSciences, cat. EK280/3-96), granzyme B ELISA kit (MultiSciences, cat. EK2173-96), and PCSK9 ELISA kit (Solarbio, cat. SEKM-0243).

### RT-PCR analysis

All the operations were carried out according to the manufacturer’ s instructions. Gene expression was normalized to β-actin and calculated with the formula 2^-ΔΔCt^. Reagent kits used are as follows: TRIzol (Vazyme, cat. R401-01), HiScript II Q RT SuperMix for qPCR kit (Vazyme, cat. R223-01), and ChamQ Universal SYBR qPCR Master Mix kit (Vazyme, cat. Q711-02/03). Primers are shown as follows: β-actin (F: AGCCTTCCTTCTTGGGTATGG; R: CAACGTCACACTTCATGATGGAAT), pcsk9 (F: GAGACCCAGAGGCTACAGATT; R: AATGTACTCCACATGGGGCAA), ifn-γ (F: CAACAGCAAGGCGAAAAAGG; R: CCTGTGGGTTGTTGACCTCAA), gzmb (F: ATCAAGGATCAGCAGCCTGA; R: TGATGTCATTGGAGAATGTCT), tnf-α (F: GCCACCACGCTCTTCTGTCT; R: GGTCTGGGCCATAGAACTGATG), perforin (F: AGCACAAGTTCGTGCCAGG; R: GCGTCTCTCATTAGGGAGTTTTT), ldlr (F: CCTCAAGTACCTTGGTATGACGC; R: GAGGCTGTCGGTCAGGATG), cd36 (F: TCGGAACTGTGGGCTCATTG; R: CCTCGGGGTCCTGAGTTATATTTTC), cd8a (F: CCGTTGACCCGCTTTCTGT; R: CGGCGTCCATTTTCTTTGGAA), and tgf-β (F: CCGCAACAACGCCATCTATG; R: CTCTGCACGGGACAGCAAT).

### Statistical analysis

Unpaired *t*-test, one-way ANOVA, or two-way ANOVA was performed for the comparison between groups. Data are presented as mean ± standard error. *p*-value < 0.05 was considered to be significant ("*" means *p*-value < 0.05 and "**" means *p*-value < 0.01 while "ns" means not statistically significant).

## Data availability statement

The original contributions presented in the study are included in the article/**Supplementary material**. Further inquiries can be directed to the corresponding authors.

## Ethics statement

The animal study was reviewed and approved by Animal Ethical Committee of School of Pharmacy Fudan University.

## Author contributions

RW, PH, and HL completed the biological and analysis experiment, and drafted the manuscript. XZ and MF conceived and proofread the manuscript. All authors approved the submitted version.

## Funding

This work was supported by the Science and Technology Supporting Project by Shanghai Municipal Science and Technology Committee (19431903000) and Shanghai Sailing Program (21YF1401900).

## Conflict of interest

The authors declare that the research was conducted in the absence of any commercial or financial relationships that could be construed as a potential conflict of interest.

## Publisher’s note

All claims expressed in this article are solely those of the authors and do not necessarily represent those of their affiliated organizations, or those of the publisher, the editors and the reviewers. Any product that may be evaluated in this article, or claim that may be made by its manufacturer, is not guaranteed or endorsed by the publisher.
