# Supplementary material for: Inhibition of PCSK9 enhances the antitumor effect of PD-1 inhibitor in colorectal cancer by promoting the infiltration of CD8+ T cells and the exclusion of Treg cells
Source: Front Immunol. 2022 Aug 8;13:947756. doi: 10.3389/fimmu.2022.947756 (PMC9393481; doi:10.3389/fimmu.2022.947756)
Supplement: Supplementary file 1 [file DataSheet_1.docx]

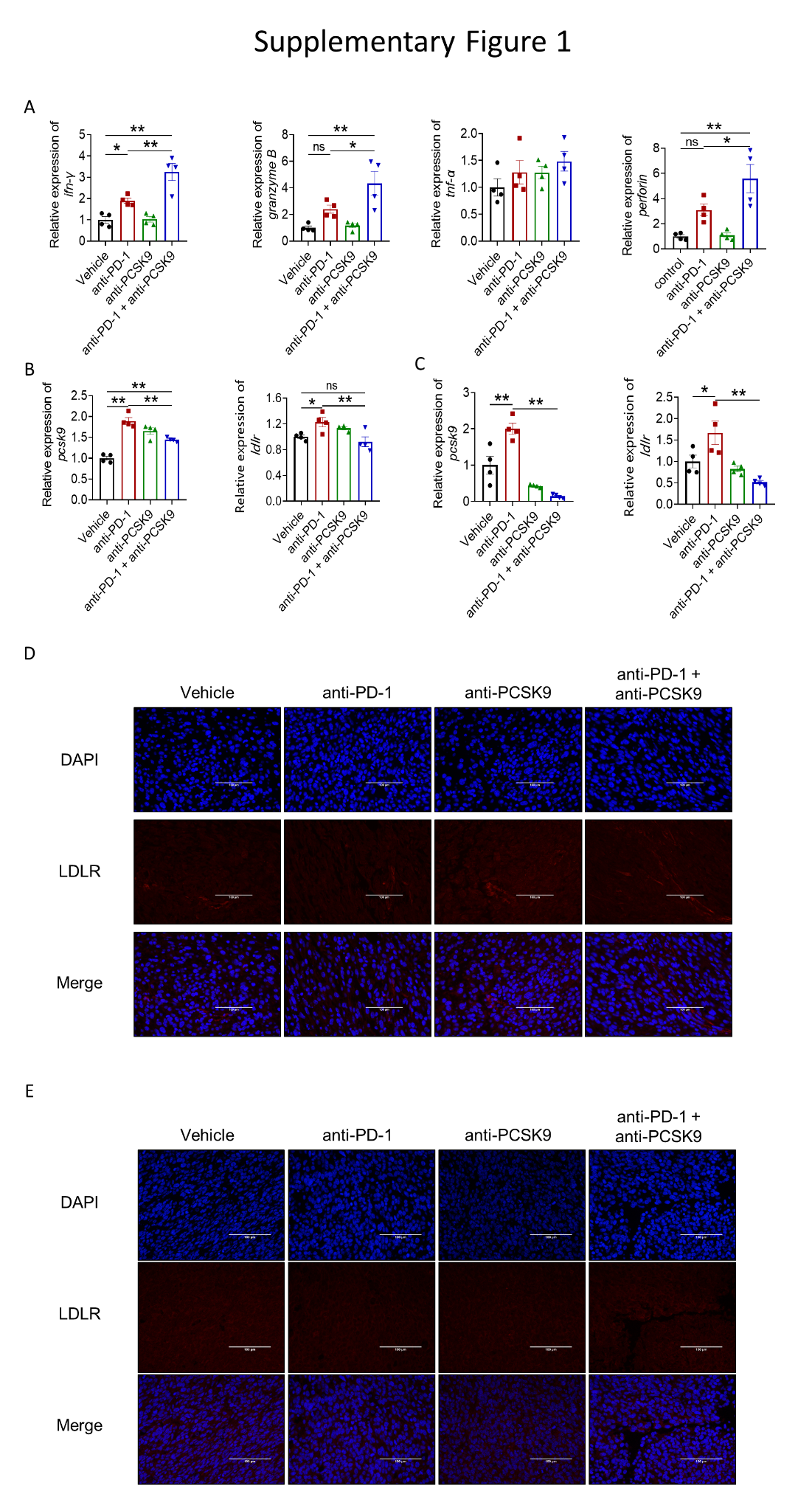


**Supplementary Figure 1 (A, B)** Analysis of IFN-γ, granzyme B, TNF-α, perforin, PCSK9 and LDLR gene expression in MC38 tumors. **(C)** Analysis of PCSK9 and LDLR gene expression in CT26 tumors. Immunofluorescence staining of LDLR in tumor of MC38 CRC model **(D)** and CT26 CRC model **(E)**


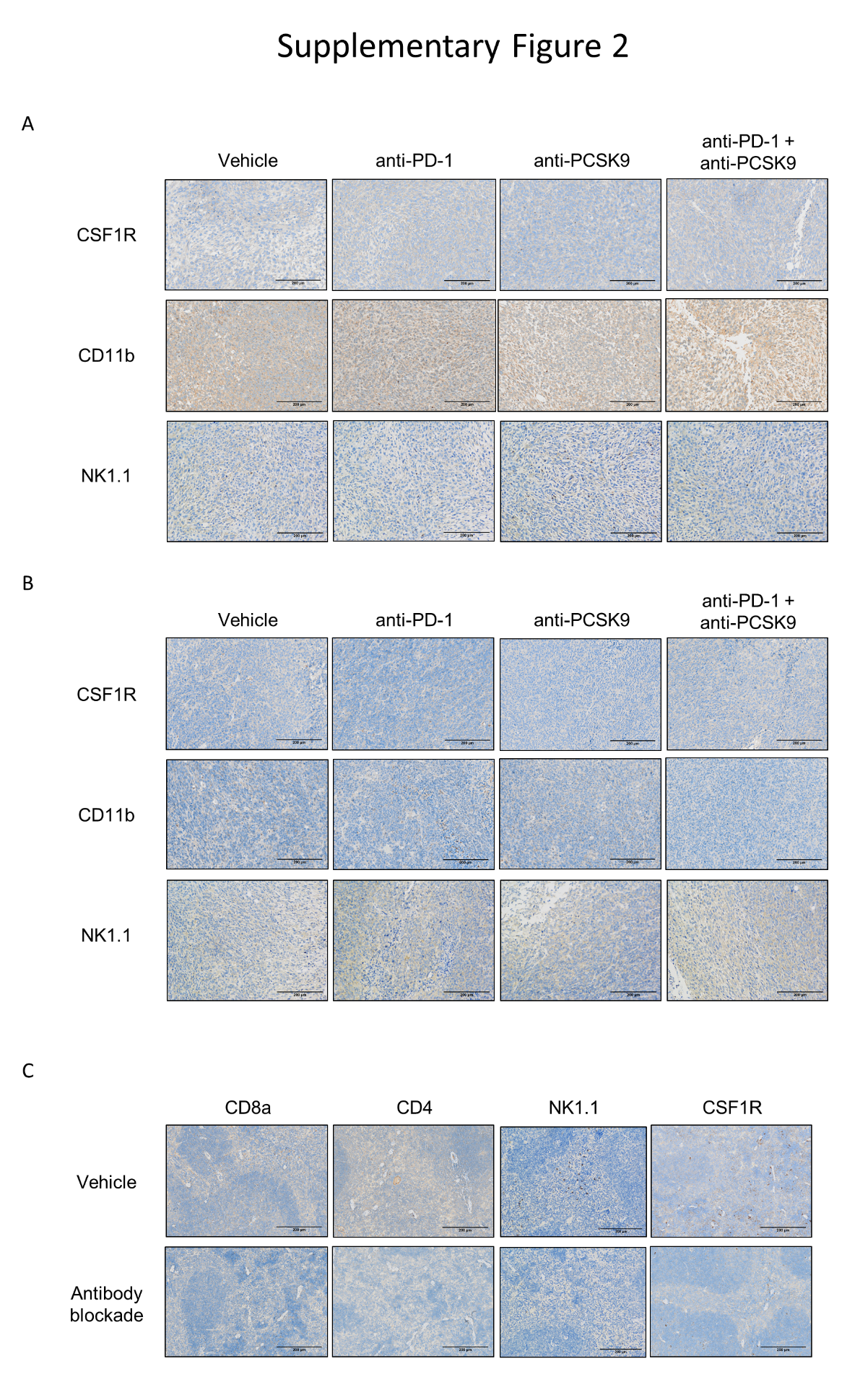


**Supplementary Figure 2** CSF1R^+^, CD11b^+^, NK1.1^+^ cells in MC38 tumors **(A)** and CT26 tumors **(B)**. **(C)** IHC staining of cells with relevant marker in spleen after antibody blockade.
